# Behaviors in Educational Settings during and after the COVID-19 Pandemic

**DOI:** 10.3390/bs13050411

**Published:** 2023-05-15

**Authors:** Xin Dong, Yi Ding

**Affiliations:** 1 Department of Teacher Education, Nicholls State University, Thibodaux, LA 70301, USA; 2 Graduate School of Education, Fordham University, New York, NY 10458, USA; yding4@fordham.edu

This editorial is an introduction to the Special Issue “Behaviors in Educational Setting”. It provides a brief overview of the research trends in the field.

Research on human behaviors in educational settings has very valuable implications for effective learning and teaching. The flourishing results of educational research on human behaviors have deepened our understanding on how to support life-span learning and development across different age groups. Research findings in early intervention areas revealed which pivotal skills are essential and socially significant for children from their early years to their later life [1,2]. Early behavioral interventions based on applied behavior analysis have been proven to have a remarkable impact on countless young children with special needs and their families [3]. Research on teacher and student behaviors in the classroom have brought new insights into how to effectively deliver instructions and improve student behavior, leading to more effective learning in school settings [4,5]. Research on life skills and employment training revealed insights into how to deliver the best training for life and employment skills for young adults with special needs, helping them to become independent and productive individuals in society [6].

In the past few years, the COVID-19 pandemic has triggered a series of changes worldwide. Virtual learning offered an alternative way for people to be able to learn and work from home [7]. Virtual technology plays a more important role in education today than ever before. As we gradually return to normal life, it is not difficult to observe new phenomena taking place for teachers and students across different age groups in educational settings. During the pandemic, we were all forced to learn a lot of information about technologies that enable us to adopt virtual learning and work in a special era [8]. Everyone has at least experienced working from home for a period of time. Some people in certain fields are still working from home. However, virtual teaching and learning is essentially different from in-person teaching. Individuals with different learning styles learn best through different approaches. Some kinesthetic learners learn best via hands-on activities. Some people understand material better when they learn and discuss in small groups. Some people rely on visual or audio inputs. Teachers in the classrooms could differentiate instructions based on each student’s learning style and their interactions in class. However, teachers in virtual mobility can only obtain limited information from the students. Virtual learning might not be effective for people with certain learning needs.

Some researchers have already conducted research on behaviors in educational settings during this special period of history [8]. This Special Issue mainly focuses on an analysis of human behaviors in an educational setting against the backdrop of the world-wide pandemic. Among the articles already published in this Special Issue, we have an article on the relationship between a principal’s leading style and teacher behaviors [9]. There is also an article focusing on parents’ perceptions of the effects of early intensive behavioral interventions for children with autism [10]. Another article conducted an analysis on the effects of motivational and behavioral factors on job productivity for academic librarians [11].

We hope we have contributed to the literature by publishing research on human behaviors in educational settings in this special period in history. The areas of interests for this Special Issue include, but are not limited to, human behaviors related to learning and teaching, social behaviors, applied and translational behavior analysis, behavioral therapy, behavioral consultation, experimental behavioral analysis, clinical behavioral analysis, behavioral training, behavioral intervention, professional training, and incidental teaching with a focus on behavioral changes in educational settings.
